# Impact of an Interprofessional Education Activity on Student Knowledge and Attitudes Regarding Patient Safety

**DOI:** 10.3390/pharmacy11020065

**Published:** 2023-03-24

**Authors:** Deepti Vyas, Tracey DelNero, Benjamin Reece

**Affiliations:** 1 Thomas J. Long School of Pharmacy, University of the Pacific, Stockton, CA 95211, USA; 2 School of Health Sciences, University of the Pacific, Sacramento, CA 95817, USA; 3 Speech Language Pathology Assistant Program, San Joaquin Delta College, Stockton, CA 95207, USA

**Keywords:** interprofessional education, patient safety, root cause analysis, pharmacy, speech language and pathology, physician assistant

## Abstract

This study aimed to measure the impact of an interprofessional education (IPE) activity on student knowledge and attitudes regarding patient safety. Two 4 h IPE activities were designed to provide students with foundational information regarding patient safety. Interprofessional teams discussed the individual curricula and roles/responsibilities of each represented health profession. Teams then served on a mock committee tasked with completing a root cause analysis of a fictitious sentinel event. Students completed a pre/post-quiz and pre/post-attitudes survey to measure knowledge and attitudes. Five months later, students reconvened to serve on a second mock sentinel event committee. Students completed a post-activity survey after the second activity. Four hundred and seven students participated in the first activity, while two hundred and eighty participated in the second activity. Quiz score comparisons revealed improved knowledge, with post-quiz scores being significantly higher. Pre- and post-attitude survey comparisons indicated a significant improvement in participant attitudes towards interprofessional teamwork. Seventy-eight percent of students reported the IPE activity enhanced their ability to “engage other health professions students in shared patient-centered care”. This IPE activity resulted in knowledge and attitude improvement related to patient safety.

## 1. Introduction

Health professionals interact and collaborate with a wide range of colleagues from other disciplines. It is imperative that university programs prepare students to engage in interprofessional practice to maximize patient outcomes [1]. The World Health Organization (WHO) states that training programs must prepare “collaborative practice-ready” practitioners to function in modern healthcare systems [2]. Interprofessional Education (IPE), defined as two or more professions working together to learn from and about each other to improve collaboration and the quality of care, is increasingly being implemented by training programs across healthcare professions as a method of preparing students to meet this challenge [2,3,4].

In 2000, the seminal report To Error is Human: Building a Safer Healthcare System by the Institute of Medicine (IOM) highlighted those factors such as poor system design, lack of teamwork and distrust between practitioners that lead to tens of thousands of preventable deaths per year in the U.S. healthcare system [5]. The follow-up report, Crossing the Quality Chasm: A New Health System for the 21st Century, offered recommendations to redesign healthcare systems to address the challenges previously identified [6]. Underlying these recommendations is the tenet of creating systems that prioritize patient safety and collaboration and teamwork between practitioners. Prioritizing safety through collaboration begins at pre-clinical training levels with increased opportunities for interdisciplinary training [6].

Over the last two decades, as health institutions work to address the systematic concerns highlighted by the IOM, IPE programing in university training programs has shown positive results for changing attitudes, increasing knowledge and developing skills in student healthcare professionals [7]. More specifically, IPE has been shown to help students understand the roles and responsibilities of their interprofessional peers, recognize overlap in their professional functions and develop positive attitudes towards other professionals [8,9,10,11]. Health profession educators have used IPE to educate students on healthcare issues common across disciplines including medical ethics, professionalism, empathy, teamwork and addressing medical errors [12,13,14,15,16,17].

Interprofessional education programming specifically to address medical errors has taken two forms; medical error disclosure and quality improvement (QI) through root cause analysis (RCA). Interprofessional education programing has been shown to be effective in teaching team-based error disclosure that reduces blame and prioritizes communication with the patient [17]. IPE programing to teach retrospective response to medical errors through a QI perspective using RCA of sentinel events has also been documented with positive results [17,18,19,20].

An error in a healthcare system that leads to either severe temporary or permanent patient harm or death is deemed a sentinel event [21]. Death or other events that are a part of the normal progression of a patient’s medical condition are not considered sentinel events. Sentinel events can occur as a result of a faulty system, improper processes, or human error. The Joint Commission requires healthcare systems to formally evaluate any sentinel event to address and rectify any weaknesses in the system. To this end, healthcare organizations are required to create an official team tasked with investigating and creating a measurable response plan with a reasonable timeline for implementation [21]. The RCA model is a common framework within the QI arena that provides a standardized process for analyzing sentinel events or patient safety events [22]. The RCA tool allows healthcare systems to identify system vulnerabilities that contributed to a sentinel event or patient safety event [19,20,23]. As future members of interprofessional teams, it is essential to introduce health profession students to this tool early on in their training. Medical error reduction and improved patient safety require that all healthcare workers engage in the surveillance and early rectification of system vulnerabilities.

When conducting an RCA, the first step is to define the problem. Once the problem is defined, the team will collect data to determine possible causes of the problem. Through interdisciplinary collaboration, the team considers each possible cause, ensuring that all team members have the opportunity for input. Through this discussion, the team defines the “true root cause leading up to the event or problem” [23]. Solutions are then brainstormed for the current problem and any system vulnerabilities. The team decides the most appropriate solution and develops an implementation plan. The final step for the team is to assess the effectiveness of the solution to determine if further action is necessary [23]. Training health profession students on key QI concepts, such as roles and responsibilities, the impact of medical errors on patient safety, interprofessional teamwork as a way to address patient safety and more specifically the components of the RCA, ensures that graduates can serve on sentinel event committees and can implement meaningful system improvements [19,20,22]. Providing this training as part of IPE can allow for a multifaceted approach and the robust discussion of improvement strategies [17,24,25]. It can also promote interprofessional equality as team members learn to respect and value each other’s perspectives [16]. Training health profession students on patient safety concepts, such as identifying root causes and working to address system vulnerabilities through sentinel event committees, can help promote future interprofessional collaboration and meaningful improvements in healthcare systems [25]. The purpose of this study was to measure the impact of participating in an IPE activity on student knowledge and attitudes regarding medical errors and QI procedures for patient safety.

## 2. Materials and Methods

The Institutional Review Board (IRB) at the University of the Pacific deemed the study exempt. Consent was waived as this was a retrospective study.

Students from the pharmacy, physician assistant (PA), physical therapy (PT) and speech–language pathology (SLP) programs participated in a two-part IPE activity focusing on patient safety. All programs required student attendance, but each program determined how students received curriculum credit. Speech–language pathology, PA and pharmacy students received points within a course, while PT participation satisfied a graduation requirement. Four hundred and seven graduate-level students participated in the first IPE event. Of the 407 participants, the professional breakdown was SLP 8% (n = 31), PA 22% (n = 89), PT 18% (n = 74) and pharmacy 52% (n = 213). Pharmacy students were all first-year professional students. Two cohorts each from the PA and PT programs participated. These students were either in their first or second year of graduate training. The SLP cohort was in its second semester of graduate training.

Ten days before the IPE activity, students completed a pre-quiz (Table 1) to measure their knowledge at baseline and a pre-attitudes survey to measure their attitudes regarding patient safety and the utility of interprofessional collaboration. The attitudes survey was a 40-item Likert scale survey adapted from Madigoski and Featherstone [26,27]. Appendix A contains the subscales retroactively developed for this study. One week before the first IPE activity, students received an online learning module that provided vital information regarding patient safety, including statistics, QI methods such as the RCA model and the development of process flow charts, fishbone diagrams and two-by-two grids to identify the best action plan to remedy system vulnerabilities. Students also received a patient case with information related to a sentinel event at a fictitious local hospital. An interprofessional task force consisting of volunteer faculty from each represented program developed the patient case. The patient case involved the death of a patient from an opioid overdose that occurred due to a breakdown in existing processes, lack of policies and human error. Each faculty member incorporated case components that would require input from their respective profession.

This first event involved forty-seven interprofessional teams. Each team consisted of 1 SLP student, 1–2 PA students, 4–5 pharmacy students and 1–2 PT students. Due to cohort size discrepancies, 11 out of 31 SLP students rotated between two teams to ensure that all teams benefited from their perspective. Eight classrooms housed the 47 teams. Each room had a faculty member to facilitate student progress and provide the activity debrief. The interprofessional teams first met to engage in a small group discussion of individual program curricula and the roles/responsibilities of each profession. Students then attended a one-hour lecture led by a QI expert from a local hospital system. The lecture highlighted the importance of a workplace culture focused on patient safety and provided some directed information on how to serve on an RCA committee, complete a critical systems analysis, develop a process flowchart, create a fishbone diagram and develop a specific and measurable action plan. Teams then served on a mock sentinel event committee and completed an RCA complete with a flow diagram, fishbone diagram and two-by-two grid. After the event, each faculty facilitator conducted a discussion involving the four to six interprofessional teams in their classroom inquiring about team responses and showed an example of a completed RCA. One week following the IPE event, students completed a post-quiz and post-attitudes survey using Google Forms.

Five months later, the same SLP, PA and pharmacy students reconvened for a second mock sentinel event. Students from the PT program were unavailable due to schedule conflicts. Prior to the second activity, students individually completed a shared mental model worksheet (Appendix B). For this second activity, seven classrooms housed 29 teams consisting of 1 SLP, 1–2 PA and 5–7 pharmacy students. Teams first completed the same shared mental model worksheet identifying, through peer teaching, the roles/responsibilities of each profession and outlining positive and negative teamwork behaviors agreed on as a team. Teams also outlined factors that would enhance their abilities to communicate and work as a team. Completing the shared mental model worksheet allowed teams to develop shared understanding or roles and responsibilities prior to engaging in more intensive problem solving. Each interprofessional team then served as a mock sentinel event committee to discuss the second patient case. The second sentinel event featured a patient who developed aspiration pneumonia due to breakdowns in process, faulty policies and human error. Once again, teams submitted a worksheet consisting of a process flowchart, fishbone diagram and two-by-two grid. All students received a faculty-developed attitudes survey five months after completing the second event. The attitudes survey consisted of eight questions regarding the impact of the IPE activity using a 5-point Likert scale (1 = Definitely worsened to 5 = Definitely enhanced).

### Statistical Analysis

Survey results from the first IPE event were collated based on the participant’s identified profession. Two scales were developed from selected items using reliability coefficients and exploratory factor analysis. To develop scales to measure attitudes towards medical errors and attitudes towards teamwork, items from the administered survey were first categorized based on the prevailing topic of each. The scoring direction was determined and reverse scoring was applied where appropriate. Items that were ambiguously worded in terms of direction were discarded. Scales were refined by eliminating items that negatively affected reliability. Using Cronbach’s alpha analysis, scale reliability testing was completed on select items from the two published surveys previously mentioned [26,27]. Exploratory factor analysis was then conducted to assess the internal consistency of each final scale. The reliability of the two scales was established through statistical analysis of the results of the survey. Cronbach’s Alpha for the Attitudes towards Medical Errors scale calculation using the pre-survey responses was 0.627 for the eight items, which is questionable. Therefore, the same analysis was conducted using the post-survey responses. The post-survey responses on items used for the Attitudes towards Medical Errors scale were 0.765, which is considered acceptable. Exploratory factor analysis showed item loading on two factors when using both the pre-survey and post-survey data. The two factors were items targeting attitudes towards issues external to oneself or the practice and items involving students rating how they might react to medical errors. Both are relevant to the single construct of Attitudes towards Medical Errors. These results suggest moderately strong internal consistency for the scale. Cronbach’s Alpha for the Attitudes towards Teamwork scale of seven items was 0.796 using the pre-survey responses and 0.802 using the post-survey responses. Exploratory factor analysis indicated loading on only one factor for both pre- and post-survey responses. These results suggest strong internal consistency. Each scale score was calculated as the sum of item scores within the scale. The resulting scales were “Attitudes towards Medical Errors” and “Attitudes towards Teamwork in Medical Settings.” The descriptive statistics of the initial sentinel event surveys were used to describe students’ knowledge and attitudes from the survey results. These scales were developed after the data were collected to determine the growth specifically in attitudes and knowledge of medical errors and teamwork. Further statistical analysis was conducted after the second IPE event using the faculty-designed survey. This analysis included descriptive statistics, including mean and percentage, to describe student attitudes more specific to the second IPE experience.

## 3. Results

Ninety-two percent (373) of the 407 students completed the pre- and post-surveys.

The survey response rates varied by discipline (SLP 94%, PA 92%, PT 84% and pharmacy 90%). The paired pre/post-quiz scores revealed improvement, albeit modest, on all questions and a higher total score, 9.08 versus 10.46 (Table 1).

Paired *T*-tests were conducted to determine if a significant difference existed between pre-survey and post-survey scale scores. The results are indicated in Table 2 and Table 3. The findings of the *t*-test analysis for the entire cohort were significant across both the Attitudes Towards Errors scale and the Attitudes Towards Teamwork scale. For SLP students, the findings of the *t*-test analysis were also significant with t(28) = −4.815, *p* < 0.001 for the Attitudes Towards Errors scale and t(28) = −5.223, *p* < 0.001 for the Attitudes Towards Teamwork scale. Similarly, the findings of the *t*-test analysis for the PA students were also significant with t (83) = −6.935, *p* < 0.001 for the Attitudes Towards Errors scale and t(83) = −4.144, *p* < 0.001 for the Attitudes Towards Teamwork scale.

Two hundred and eighty (twenty-nine SLP, forty-three PA, two hundred and eight pharmacy) students participated in the second IPE activity. A total of 87% (N = 244, RR: SLP 66% (N = 19), PA 95% (N = 41), pharmacy 88% (N = 184)) completed the attitudes survey after the activity. At least 72% of all students reported enhanced (somewhat or definitely) attitudes to each survey item (Table 4).

A total of 78% reported the IPE activity enhanced their ability to “engage other health professionals in shared patient-centered care” (N = 217. Mean = 4.27/5; SLP 74% (n = 14), PA 97% (n = 40), pharmacy 88% (n = 163)). A total of 75% reported that the IPE activity enhanced their “understanding of the clinical approach and perspective of various health professions” (N = 211, mean 4.19/5; SLP 84% (n = 16), PA 95% (n = 39), pharmacy 85% (n = 156)).

Each profession reported enhanced ability to ‘implement effective team attributes to reduce medical errors” (N = 212, 76%, mean = 4.19/5; SLP 58% (n = 11), PA 98% (n = 40), pharmacy 87% (n = 161)). It should be noted that the number of participants refers to the number of students that participated in the study, which is lower than the number of students who participated in the IPE programming. An increased mutual respect was noted by 77% of the students overall (N = 215, mean = 4.26/5; SLP 74% (n = 14), PA 100% (n = 41) and pharmacy 87% (n = 160)). A total of 76% (N = 214) of respondents reported increased knowledge of the various health professions’ scope of practice and 79% overall (N = 222, mean = 4.21) reported enhanced “ability to apply your professional knowledge and that of other professions in providing patient care” (SLP 79% (n = 15), PA 97% (n = 40) and pharmacy 91% (n = 167)). A total of 78% of students reported the IPE event enhanced (somewhat or definitely) their “confidence to include various health profession during future clinical or professional practice to enhance patient care” (N = 217, mean = 4.25/5; SLP 74% (n = 14), PA 97% (n = 40) and pharmacy 88% (n = 163)). A slight enhancement of patient safety knowledge was noted overall, as 72% reported enhanced “knowledge of patient safety and QI concepts” (N = 202, mean = 4.04/5; SLP 63% (n = 12), PA 93% (n = 38) and pharmacy 82% (n = 152)).

## 4. Discussion

This study showed positive results for knowledge and attitudes by all health profession students, reinforcing the previous literature such as Marshall and colleagues’ significant findings in the improvements in teamwork and interprofessional attitudes through medical error simulation [18]. In terms of the pre- and post-quiz targeted knowledge acquisition related to the process of RCA, however, while there was growth in all cohorts from the pre-quiz to the post-quiz, it was quite modest. In comparison, the pre- and post-surveys targeted a more global knowledge of medical errors and teamwork in medical settings. Statistical analysis of the survey results indicated a significant change in student attitudes towards these two constructs. Across all surveys, a majority of students reported significant benefits from each IPE activity. The probability analysis of attitude data from the first sentinel event revealed statistically significant improvement (*p* < 0.001) in student attitudes towards medical errors and working with interprofessional healthcare teams. Student attitudes toward collaborative interprofessional practice indicated improved understanding of the perspectives and scope of healthcare team members after the second IPE event. At least three-quarters of all students surveyed reported benefit from the event across seven of the eight categories assessed, except for the category of “knowledge of patient safety and QI” for which 72% indicated benefit. The lower percentage of reported benefit for this particular item may be due to the fact that this IPE program was their first exposure to strategies for addressing patient safety and QI. The PA students reported the most significant benefit from the second event, with over 97% noting knowledge enhancement for all categories (mean = 4.23/5 to 4.55/5). At least 82% of pharmacy students also reported perceived benefit, with mean item scores of 4.04/5 to 4.22/5. The SLP students’ attitudes were varied, with a standard deviation of 8.29. The SLP students rated “understanding of the clinical approach and perspective of various health professions” the most beneficial, with 84% of SLP students reporting enhanced knowledge. Fewer SLP students reported benefits regarding implementing effective team attributes to reduce medical errors (58%, mean = 3.74/5) and patient safety and QI concepts (63%, mean = 3.63/5). The etiology for the attitude discrepancy between the PA and SLP students is unknown due to the lack of qualitative student feedback. Individual team dynamics may play a role, but it may also be that the students gained significant knowledge through the first event and that additional training on QI concepts was not beneficial for them. For the SLP students, the relatively smaller cohort size may have contributed to the difference in results when compared to pharmacy students.

This study has several limitations. Medical students were not included in the IPE event as the university does not have a medical school. Since physicians are important stakeholders on patient safety teams, the addition of medical students would have led to richer discussion. Cohort size disparities resulted in a disproportionate number of pharmacy students in the teams compared to the other professions. This discrepancy may have resulted in less engagement from some pharmacy students. Having some of the SLP students shared between two teams for the initial event may have impacted team dynamics, as there would not be SLP input for all aspects of the discussion. The discrepancy in participants was further exacerbated by a smaller response rate to the survey after the second IPE event from the SLP students. The response rate from the SLP students was 66% as compared to 95% from PA students and 88% from pharmacy students. Another limitation related to cohorts was that the PT students were unavailable to participate in the second IPE activity. The inclusion of PT students in the second event could have led to more robust discussion especially in the area of process improvement. Future iterations will include PT students, if possible. In addition, this study did not use a validated interprofessional attitudes survey such as the Student Perceptions of Physician–Pharmacist Interprofessional Clinical Education revised instrument (SPICE-R) or the Interprofessional Collaborative Competencies Attainment Survey. The use of a validated survey would have provided further evidence of improvement in interprofessional attitudes. This deficiency will be rectified in future renderings of these events by administering the SPICE-R survey to measure interprofessional attitudes. Due to an error after the second event, SLP students did not complete the immediate post-survey and only completed the 5-month mark, which may have affected student attitudes regarding the IPE activity. In addition, this study did not review the accuracy of team submissions, which would have provided information on the quality of the work submitted by interprofessional teams. Evaluating the accuracy of team submissions could have helped identify gaps in student skills and provided more information on areas that require reinforcement. The qualitative analysis of teamwork submissions will be the focus of future research. Future research will investigate the impact that exposure to IPE events focused on QI and medical errors has on clinical practice and patient safety upon graduation.

## 5. Conclusions

Patient safety is the responsibility of all healthcare professionals [1]. The early education of students on key patient safety concepts and QI techniques can ensure that graduates are competent and may help prepare them to take on leadership roles in their institutions [22]. Early exposure to interprofessional teamwork is also likely to impact patient safety positively once students begin clinical practice [21]. Physician assistants, pharmacists, PTs and SLPs are integral members of the healthcare system and have a significant role in managing hospitalized patients. The nuances of acute care require that all team members are aware of patient safety concerns and can identify systems issues that leave the institution vulnerable to patient harm. To ensure engagement by all professions, the mock sentinel events included systems breakdowns related to each represented profession. The significant improvement in student attitudes and knowledge was encouraging and demonstrated that this approach was successful. For other programs looking to enhance their IPE curriculum, it is important to create authentic experiences that simulate actual clinical practice. This IPE program replicated an important process in the patient safety arena and as such provided the opportunity for discussion about the root causes of medical errors and the role that each profession has in mitigating errors and patient harm. This starts with understanding the roles and responsibilities of each profession, understanding that each profession is responsible for identifying and reporting patient safety concerns and that a team-based approach is needed in mitigating patient safety issues. This is a powerful strategy in building future health professionals.

## Figures and Tables

**Table 1:** Pre/post-Knowledge Quiz Results.

Quiz Question ^a^	Pre-Mean	Post-Mean
Which are likely root causes of the error?	0.42	0.67
How would you categorize this error?	0.68	0.72
How are fishbone diagrams used in hospital settings?	0.72	0.96
Identify the false statement below	0.75	0.89
Of the following, which are possible corrective actions for this case?	0.76	0.90
Which of the following opioid medications is the most potent?	0.77	0.87
Choose the correct definition for the phrase “near-miss” error.	0.79	0.91
What is the definition of root cause analysis?	0.79	0.88
What is the final step of root cause analysis?	0.82	0.83
A medical error was recently discovered at your hospital. What should the hospital do after they have implemented steps to prevent future errors?	0.83	0.94
To maximize the effectiveness of a root cause analysis you should only involve experts in the field.	0.84	0.96
What does root cause analysis aim to prevent?	0.91	0.95
Total score	9.08	10.46

^a^ Each question scored as 1 for correct response or 0 for incorrect response. These were paired to each individual student.

**Table 2:** *T*-tests comparing cohort means of scales from pre-test and post-test surveys.

n = 373	Pre-Test		Post-Test		t(372)	*p*
	M	SD	M	SD		
Attitudes Towards Errors	31.457	3.000	33.311	3.443	−10.892	0.000
Attitudes Towards Teamwork	26.472	3.729	28.622	3.448	−12.949	0.000

These were paired to each individual student.

**Table 3:** *T*-tests comparing means of scales from pre-test and post-test surveys for the Pharmacy students.

n = 194	Pre-Test		Post-Test		t(193)	*p*
	M	SD	M	SD		
Attitudes Towards Errors	31.340	3.040	32.871	3.647	−6.287 *	0.000
Attitudes Towards Teamwork	25.639	3.447	27.768	3.539	−8.835 *	0.000

* These were paired to each individual student.

**Table 4:** Student attitudes regarding the second interprofessional education event.

Statement: Rate the Impact of the IPE Activity on Your ^1^	Overall Mean (SD) N = 244 (RR = 87%)	SLP Mean (SD) N = 19 (RR = 66%)	PA Mean (SD) N = 41 (RR = 95%)	Pharmacy Mean (SD) N = 184 (RR = 88%)
Ability to engage other health professionals in shared patient-centered care	4.27 (0.65)	4.11 (0.79)	4.55 (0.54)	4.22 (0.65)
Understanding of the clinical approach and perspective of various health professions	4.19 (0.65)	4.11 (0.64)	4.52 (0.59)	4.12 (0.64)
Ability to implement effective team attributes to reduce medical errors	4.19 (0.69)	3.74 (0.86)	4.40 (0.53)	4.19 (0.64)
Developing a climate of mutual respect among varied health professionals	4.26 (0.71)	4.05 (0.89)	4.57 (0.49)	4.21 (0.71)
Knowledge of risk management and quality improvement concepts	4.04 (0.66)	3.63 (0.67)	4.23 (0.57)	4.04 (0.66)
Knowledge of various health professions’ scope of practice	4.22 (0.65)	4.05 (0.6)	4.55 (0.59)	4.20 (0.64)
Ability to apply your professional knowledge and that of other professions in providing patient care	4.21 (0.59)	4.05 (0.69)	4.40 (0.5)	4.19 (0.66)
Confidence to include various health professions during future clinical or professional practice to enhance patient care.	4.25 (0.64)	4.05 (0.76)	4.5 (0.54)	4.21 (0.58)

^1^ Survey based on a Likert scale of 1–5 with 1 = Definitely worsened to 5 = Definitely enhanced.

## Data Availability

Data are available upon request.

## Appendix A

Attitude towards Medical Errors Scale, Attitude towards Teamwork Scale [26,27].

Attitudes towards Medical Errors					
1. Making errors in healthcare delivery is inevitable	1	2	3	4	5
2. Only physicians can determine the cause of a medical error	1	2	3	4	5
3. If there is no harm to a patient, there is no need to address a medical error	1	2	3	4	5
4. If I saw a medical error, I would keep it to myself	1	2	3	4	5
5. Errors that reach the patient should be reported even if the patient is not harmed	1	2	3	4	5
6. I would report an error that was caught and corrected before affecting the patient	1	2	3	4	5
7. When something does not seem right about the patient’s care, I would ask questions of any experienced member of the team regardless of their authority	1	2	3	4	5
8. I feel confident analyzing a case to find the cause of an error	1	2	3	4	5
9. I am confident in reacting to patient safety concerns	1	2	3	4	5
Attitudes towards Teamwork					
1. I feel confident working with a team, mapping out the follow of care process	1	2	3	4	5
2. I feel confident when working as part of a multidisciplinary team	1	2	3	4	5
3. I like to collaborate with other healthcare professionals	1	2	3	4	5
4. I am comfortable communicating with individuals from other healthcare professions	1	2	3	4	5
5. Working in a multidisciplinary team would make me feel anxious	1	2	3	4	5
6. I feel at ease working as part of a team of individuals from my own profession	1	2	3	4	5
7. I find that cooperation and communication with individuals from other professions can be difficult	1	2	3	4	5
Survey based on a Likert scale of 1–5 with 1 = Definitely worsened to 5 = Definitely enhanced.					

## Appendix B. Shared Mental Model Contract

As a team, getting on the same page is a central responsibility of each team member. As you think about your role within the healthcare system, which of the following are your professional responsibilities?

Put an X by each area that you think is the role/responsibility of each profession in a hospital.

Please explain to the team why this is the responsibility of your profession.

**Responsibility**	**PA**	**PharmD**	**SLP**
Diagnosis			
Assessment of the patient’s condition			
Developing a treatment plan			
Putting in new ‘orders’ for the patient			
Ensuring the medication plan is appropriate			
Ensuring the monitoring plan is appropriate			
Assessing and monitoring any swallowing pathologies			

The following are considered attributes of high-functioning teams:

**Teamwork Skills**	**Definitions**	**Examples of Markers of Good Behavior**	**Examples of Markers of Poor Behavior**
Coordination	Managing synchronous and/or simultaneous activities to align the pace and sequencing of others’ contributions with goal accomplishment	Confirms roles and responsibilities of team members	Does not involve team in task
Information exchange	Giving and receiving the knowledge and data necessary for team coordination and task completion	Gives situation updates/reports key events	Fails to express concerns in a clear and precise manner
Use of authority	Observable behavior of leading the team and/or the task (as required) or accepting a non-leading role when appropriate	Gives clear orders to team members	Does not allow others to put forward their case
Assessing capabilities	Providing physical, cognitive and emotional help to team members and seeking help from others when necessary	Notices that a team member does not perform task to expected standard	Does not pay attention to the performance of other members of the team
Supporting behaviors	Providing physical, cognitive and emotional help to team mates and seeking help from others when necessary	Anticipates when colleagues will need equipment or information	Asks for information at difficult/high-workload time for someone else

Teams will work more efficiently and effectively if all members of the team are “on the same page.” Please complete the teamwork attributes worksheet below as a team, outlining what you consider “markers of good and poor behavior”.

**Teamwork Skills**	**Definitions**	**Markers of Good Behavior**	**Markers of Poor Behavior**
Coordination	Managing synchronous and/or simultaneous activities to align the pace and sequencing of others’ contributions with goal accomplishment		
Information exchange	Giving and receiving the knowledge and data necessary for team coordination and task completion		
Use of authority	Observable behavior of leading the team and/or the task (as required) or accepting a non-leading role when appropriate		
Assessing capabilities	Providing physical, cognitive and emotional help to team members and seeking help from others when necessary		
Supporting behaviors	Providing physical, cognitive and emotional help to team-mates and seeking help from others when necessary		
